# Prediction scale of response to liraglutide therapy as the method for increase of treatment efficacy in type 2 diabetes

**DOI:** 10.2144/fsoa-2021-0070

**Published:** 2022-01-31

**Authors:** Anna A Mosikian, Tatiana I Golikova, Mariia V Martjanova, Alina Y Babenko

**Affiliations:** 1 Institute of Endocrinology, Laboratory of Diabetology, World-Class Research Centre for Personalized Medicine, Almazov National Medical Research Centre, Saint Petersburg, Russia

**Keywords:** diabetes mellitus, glucagon-like peptide-1 receptor agonists, liraglutide, obesity, predictors

## Abstract

**Background:**

The effects of liraglutide on body weight and hemoglobin A1C (HbA1c) level vary greatly. The cost of this drug negatively affects treatment adherence.

**Aim:**

To reveal the baseline patient characteristics, associated with a better response to liraglutide.

**Materials and methods:**

A total of 41 patients with BMI of 39.63 ± 7.59 kg/m^2^ who received liraglutide injection up to 1.8 or 3.0 mg/day for 6 months were enrolled. Demographic and anthropometric data, parameters of glycemic control, food intake, hormones and responses to the eating behavior questionnaire were collected.

**Results:**

Weight reduction was dose-dependent (p = 0.007). Liraglutide was not effective in patients with BMI >45 kg/m^2^. The baseline HbA1c level was a significant factor for HbA1c reduction. Lower leptin and higher glucagon-like-peptide 1 concentrations might predict better weight loss response to liraglutide.

**Conclusion:**

Drug-specific efficacy predictors were assumed; thus, further studies are needed to prove their significance.

## Plain language summary

The objective of this study was to identify the baseline patient characteristics as a predictors associated with a better response to liraglutide. It is related to different effect of liraglutide on body weight and hemoglobin A1c (HbA1c) in different patients. We found that weight reduction was dose-dependent (p = 0.007) and liraglutide was not effective in patients with BMI >45 kg/m^2^. The baseline HbA1c level significantly correlated with HbA1c reduction. Lower leptin and higher glucagon-like peptide-1 concentrations might predict better weight loss response to liraglutide.

In the Russian Federation, the incidence rates of obesity among men and women aged 25–64 years are 26.9 and 30.8%, respectively and the incidence of type 2 diabetes mellitus (T2DM), according to the NATION study, is 5.4% of the population [1]. According to the T2DM registry, as of 1 January 2019, the average incidence is 2885.7 per 100,000 population [2]. Obesity is the leading etiological factor in T2DM pathogenesis. With the increase in BMI, the risk of T2DM linearly increases: with BMI >40 kg/m^2^ in people aged <55 years, the risk of T2DM increases 18.1-times in men and 12.9-times in women (compared with the risk in people with BMI of 25–30 kg/m^2^ (normal weight) and the incidence of T2DM in people aged <55 years exceeds three–four-times the values in men and women with normal body weight [3]. According to the DIRECT study, the normalization of body weight or its decrease by ≤10 kg in patients with a short history of T2DM (up to 6 years), leads to T2DM remission in 64% of the cases [4].

Obesity is the factor increasing the risk of not only T2DM but also arterial hypertension, ischemic heart disease and myocardial infarction, which leads to reduced working capacity, early disability and short lifespan [5]. Moreover, in patients with T2DM, obesity is the factor worsening the possibility to achieve compensation of carbohydrate metabolism and leads to a lower quality of life as obesity progresses [6]. Therefore, the reduction of body weight is a high-priority task, as resolving obesity will reduce the risk of T2DM and its early implementation causes possible remission in the early stages, significantly improve metabolic control and quality of life in patients with chronic T2DM and reduce cardiovascular risk.

Consequently, most patients need complementary drug therapy, which is effective for both the reduction of body weight and improvement of glycemic parameters and the correction of poor eating behaviors [7]. Among drugs from the group of glucagon-like-peptide-1 receptor agonists (GLP-1RA), liraglutide is one of the most effective weight loss medication. According to literature data, 50–63% of patients experienced weight loss and reduced hunger while taking liraglutide [7,8]. Liraglutide supplementation and compliance to lifestyle recommendations lead to a clinically significant weight loss up to 13.5%, glycemic improvement (hemoglobin A1c [HbA1c] decrease by 0.9–2.2% within 6 months of treatment) and correction of other cardiometabolic parameters systolic blood pressure and level of atherogenic lipids [9,10].

Meanwhile, the response to GLP-1RA therapy is highly variable. Thus, HbA1c decrease during GLP-1RA therapy varies from 0.9 to 2.2% in patients with T2DM and obesity. In the meta-analysis by Esposito *et al.* the proportion of participants achieving HbA1c level <7% was 47% of those taking liraglutide [11].

Nowadays, data on the presence of response predictors to GLP-1RA has accumulated. Thus, according to the ReaL study, liraglutide is more effective in glycemia reduction in patients with poorer baseline glycemic control, which is quite an expected glucose-dependent effect of the drugs [12,13]. Glucose-reducing effect was also more prominent if liraglutide was administered alone [14]. Negative predictors (poor treatment response) are glutamic acid decarboxylase antibodies, chronic diabetes and C-peptide level <0.25 nmol/l [14,15].

Meanwhile, weight loss during liraglutide therapy is associated with other predictors. According to Ryder *et al.*, glycemic response does not depend on body weight [16], but its more significant decrease in the study, as in our earlier study [13], was found in patients with grade ≥2 obesity. Moreover, we have noted a direct relationship between weight loss and glucose-dependent insulinotropic peptide (GIP) level and adequacy of ghrelin suppression after food intake [13]. The effects of GLP-1RA on weight differ with eating behaviors. Some investigators reported that patients with emotional [17] and/or external [18] eating behaviors are less sensitive to central GLP-1RA effects than patients with restrictive eating behavior. Our data revealed that patients with restrictive eating behavior tend to lose weight more than patients with two or three types [13]. In addition, we noted a correlation between the efficacy of GLP-1RA therapy and visual analogue scale (VAS) hunger intensity [13]. Another possible predictor of weight reduction might be the amount of leptin, produced by adipose tissue and regulating appetite. Under normal physiological conditions, leptin provides the onset of satiety, reducing calorie intake, has a glucose-lowering effect and reduces ectopic fat accumulation through central and peripheral mechanisms, which should have a beneficial effect on metabolic health [19].

A definite relationship was also observed between glycemic response and weight loss. In our study, among patients with HbA1c reduction of 1–2% and more, 86% lost weight by ≥5%. Moreover, among those who reported HbA1c reduction for >1%, only 26% achieved the targeted weight loss. A group of French investigators stated that double responders (by HbA1c and body weight) are young patients with acute T2DM and high BMI [13].

In recent years, predictive scales are widely used to optimize the treatment of various diseases. Following our literature review, no study has proposed predictive scales of the high efficiency of liraglutide therapy. Thus, for this drug group, determining the target group of patients who can benefit from it despite its high cost is necessary. Therefore, this study aimed to reveal the baseline patient characteristics associated with a better hypoglycemic and weight reduction response to liraglutide.

## Research design & methods

The study included 41 patients who were obese with T2DM or prediabetes receiving liraglutide at 3.0 mg/day (34%) or by the standard scheme for liraglutide therapy for T2DM with dose titration not over 1.8 mg/day (66%). The following data were collected for all patients: gender, age, body weight and BMI (body weight, kg/[height, m]^2^), fasting glycemia index, glycated hemoglobin value, insulin, C-peptide, GLP-1 and GIP concentrations in the blood, leptin level in the blood, type of eating behavior (restrictive, external and emotional) and assessment of hunger/satiety on a VAS.

The patients were divided into T2DM group and non-T2DM group, responders and non responders. Serum levels of insulin and C-peptide were measured with a fully automated biochemical analyzer (Cobase 411, Roche Diagnostics, Switzerland) with commercial sets for insulin (Insulin Elecsys, Cobas e; measurement range: 1.39–6945 μIU/ml; normal value: 17.8–173.0 pmol/) and C-peptide (C-peptide Elecsys; normal value: 0.78–1.89 ng/ml). The conversion factor for insulin is pmol/l × 0.144 = μU/ml.

Plasma levels of GLP-1 and GIP and serum level of leptin were measured with ELISA using an automatic analyzer (Manometer Model 680, BioRad, CA, USA).

The commercial set for GLP-1 (BCM Diagnostics, CA, USA) has a measurement range of 0.0–25.0 pg/ml and sensitivity of 0.04 pg/ml. The commercial ELISA Kit for GIP (Gastric Inhibitory Polypeptide, Cloud-Clone Corp, TX, USA) has a measurement range of 61.7–5000 pg/ml and sensitivity of <23.9 pg/ml. The measurement range of the commercial set for leptin (ELISA, Diagnostics Biochem Canada) is 2.0–11.0 ng/ml, with a sensitivity of 0.5 ng/ml.

The type of eating behavior was determined using the Dutch Eating Behavior Questionnaire. The questionnaire contains items related to the three scales corresponding to the three types of eating behaviors. The quantitative value by the scale is equal to the mean arithmetic of the scale scores. As normal values, the average scores were <2.4, 1.8 and 2.7 points for the restrictive, emotional and external types of eating behaviors, respectively. Values exceeding the margins were accepted as the deviation of one or several types of eating behaviors.

Answers to appetite questions were evaluated by the VAS. In the morning, strictly fasting patients answered questions, ‘How sated do you feel?’, ‘How much do you want to eat?’ and ‘How much food could you eat now?’ and participants selected a value from 1 (‘not much’/‘a little bit’) to 10 (‘a lot of’/‘very much’).

Data were collected prior liraglutide therapy and in 6 months of therapy. According to the measurements of body weight and glycated hemoglobin level in 6 months, all patients were divided as responders and non responders. Responders included patients whose body weight and glycated hemoglobin level were reduced by ≤7% from baseline and ≥1%, respectively. All collected data, excluding parameters directly showing treatment response, were considered factors associated with a greater likelihood that the patient may experience weight loss or may have decreased glycated hemoglobin levels.

Responders and non responders were compared by the abovementioned parameters with the Mann–Whitney U-test (quantitative variables) and Chi-square test (qualitative variables; with Yates’s correction for continuity – if the value in one of the wells of the fourfold system is ≤5). To evaluate the sensitivity and specificity of factors in relation to response prediction, receiver operating characteristics (ROC) curves were plotted. To evaluate the contribution of factors to the dynamics of weight loss or decrease in glycated hemoglobin level, regardless of crossing the ‘responder–non responder’ margin, analysis of variance was used. Moreover, if it was necessary to determine the linear regression coefficient and correlation coefficient, the linear regression model was used. Analyses of treatment efficacy in relation to body weight and glycated hemoglobin were performed in all patients (n = 41) and in patients with T2DM (n = 27), respectively.

## Results

The parameters of the enrolled patients are provided in Table 1 (mean ± average deviation). The parameters associated pathogenetically with T2DM are presented for all enrolled patients and patients with T2DM only.

**Table 1.** Characteristics of all participants included in the study and dynamics of the studied parameters during therapy with liraglutide.

	Prior therapy	n	p-value	In 6 months of therapy	n	p-value
Age, years (no T2D) Age, years (T2D)	43.71 ± 13.67 53.33 ± 8.64	14 27	0.022	-	14 27	
Gender, % of women (no T2D) Gender, % of women (T2D)	57.1 66.7	14 27	0.548	-	14 27	
Body weight, kg (no T2D) Body weight, kg (T2D)	118.48 ± 32.42 116.50 ± 29.71	14 27	0.912	107.25 ± 29.91 111.22 ± 29.50	14 27	0.573
BMI, kg/m^2^ (no T2D) BMI, kg/m^2^ (T2D)	38.72 ± 7.37 40.66 ± 7.75	14 27	0.438	35.12 ± 8.18 38.94 ± 7.71	14 27	0.059
Fasting glycemia, mmol/l (no T2D) Fasting glycemia, mmol/l (T2D)	5.60 ± 1.19 8.68 ± 2.31	14 13	<0.001	5.19 ± 0.66 6.77 ± 1.36	14 11	<0.001
Glycated hemoglobin, % (no T2D) Glycated hemoglobin, % (T2D)	5.57 ± 0.50 8.08 ± 1.27	14 26	<0.001	5.38 ± 0.58 7.36 ± 1.13	14 24	<0.001
Insulin concentration in blood, pmol/l (no T2D) Insulin concentration in blood, pmol/l (T2D)	223.59 ± 102.53 194.83 ± 146.90	11 8	0.456	120.36 ± 66.09 117.77 ± 73.47	10 6	0.892
Blood level of C-peptide, ng/ml (T2D)	3.68 ± 1.80	8	NA	4.11 ± 2.28	6	NA
GLP-1 concentration in blood, pg/ml (no T2D) GLP-1 concentration in blood, pg/ml (T2D)	4.36 ± 3.76 1.33 ± 2.99	13 24	<0.001	5.36 ± 3.78 0.67 ± 1.74	13 19	<0.001
GIP concentration in blood, pg/ml (no T2D) GIP concentration in blood, pg/ml (T2D)	229.25 ± 211.60 318.46 ± 156.56	13 18	0.146	299.34 ± 168.77 403.29 ± 138.15	13 14	0.126
Blood level of leptin, ng/ml (no T2D)	86.28 ± 40.95	12	NA	48.03 ± 30.04	12	NA
Type of eating behavior, % Restrictive (no T2D) Restrictive (T2D) External (no T2D) External (T2D) Emotional (no T2D) Emotional (T2D)	78.6 100.0 85.7 50.0 100.0 50.0	14 18 14 18 14 18	0.073 0.061 0.002	50.0 94.4 71.4 38.9 78.6 44.4	14 18 14 18 14 18	0.010 0.087 0.075
Answers to appetite questions (only no T2D) How sated do you feel? How much do you want to eat? How much food could you eat now?	3.93 ± 3.47 8.14 ± 2.41 7.64 ± 2.90	14	NA	6.93 ± 3.71 5.07 ± 3.29 4.79 ± 3.47	14	NA

NA: Not applicable; T2D: Type 2 diabetes.

### Factors associated with greater weight-reducing efficacy

The significant factors associated with treatment response in the T2DM and non-T2DM group were as follows:

-  Treatment group (patients taking liraglutide at 3.0 mg/day were more frequent responders; p = 0.007) reflected the dose-dependent therapeutic effect
-  The body weight before the treatment (the average body weight in responders [102.78 kg] was lower than those of the non responders [121.86 kg; p = 0.026])
-  Fasting glycemia before the treatment (mean fasting glycemia values in responders [6.00 mmol/l] was lower than that of non responders [7.94 mmol/l]; p = 0.028)

Nevertheless, if correction was made for the treatment group (analysis of variance), the baseline body weight (p = 0.317) and fasting glycemia (p = 0.607) were not significant factors.

#### Body mass index

The pretreatment BMI was not significantly different between the responders and non responders.

Based on studies regarding the description of the positive correlation between the decrease in BMI and its baseline value with GLP-1RA therapy, we constructed ROC curves of the relationship between the present response and baseline BMI.

The area under the ROC curve was 0.318 ± 0.084, with CI of 0.1525–0.4825 (p = 0.030). Therefore, we can say that BMI is a significant factor in the prediction of response to GLP-1RA therapy. However, the direction of the curve corresponds to the obtained data that patients with lower body weight (and lower BMI) respond better to the therapy.

**Figure 1.**
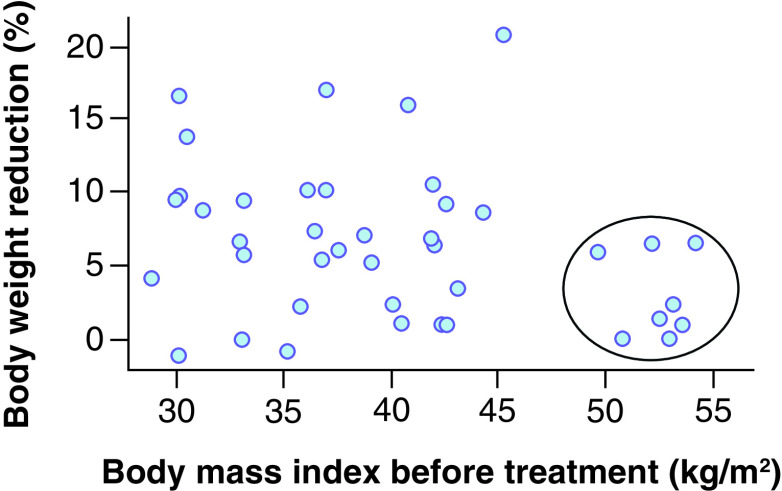
Relationship between percentage of body loss and baseline BMI value prior to treatment.

As the finding contradicted the literature data, additional analysis by the parameter was performed. Figure 1 shows the cluster of patients who have not responded to GLP-1RA therapy, in other words, patients with BMI >46 kg/m^2^.

The linear regression model based on the data of patients with BMI <46 kg/m^2^ presented a positive linear coefficient (both with and without regard to liraglutide dosage); however, the correlation based on our data were not significant (p = 0.570 with regard to liraglutide dosage; p = 0.863 without regard to liraglutide dosage).

Based on the obtained data and previously published data, the conclusion appears appropriate that GLP-1RAs can be effective in patients with BMI 30–45 kg/m^2^; however, it may be impossible to identify smaller BMI changes related to high treatment efficacy.

#### GLP-1

The percentage of weight loss correlated strongly to baseline GLP-1 value (p = 0.030; linear coefficient: 0.64), in other words, GLP-1 increase by 1 ng/ml was associated with additional weight loss by 0.64% (correlation coefficient: 0.41%). Therefore, with regard to values of the intercept (5.07) and linear correlation coefficient (0.64), the threshold value of GLP-1 in predicting the body weight loss by 7% was 3 ng/ml.

Meanwhile, the non-T2DM group had a higher GLP-1 value than the T2DM group (4.36 ± 3.76 vs 1.42 ± 3.02; p = 0.0006) before the treatment. The analysis of the correlation between the percentage of body weight loss in the T2DM group and baseline GLP-1 value showed a not significant negative correlation (linear coefficient: -0.44; p = 0.394), and in the non-T2DM group, a positive significant correlation was found (linear coefficient: 0.84; p = 0.031); furthermore, the correlation coefficient was 0.60.

Therefore, baseline GLP-1 level can be considered a predictor of response to liraglutide therapy (3.0 mg/day) in patients with obesity. With regard to the intercept (5.19) and linear coefficient (0.84), the threshold GLP-1 value of predicting body weight loss by 7% was 2.15 ng/ml. Data obtained in the common group are less informative because of group heterogeneity.

#### GIP, insulin & C-peptide

The GIP level was not significant in the comparison of responders and non responders (p = 0.373) or evaluation of correlation with weight loss (p = 0.409 without regard to T2DM; p = 0.375 with regard to T2DM).

The insulin level was not significant in the comparison of responders and non responders (p = 0.395) or evaluation of correlation with weight loss (p = 0.372 without regard to T2DM, p = 0.333 with regard to T2DM).

The C-peptide level was determined only in patients with T2DM. The factor was not significant in either comparison of responders and non responders (p = 0.500) or evaluation of correlation with body weight (p = 0.844).

Meanwhile, patients with C-peptide <0.25 nmol/l were not included into the common group, which does allow making final conclusions on the significance of its predictor. In this case, the limited data available for the analysis and the necessity of further observations should be highlighted.

#### Leptin

The level of leptin in the blood was measured only in the non-T2DM group. This level was lower in responders. The linear model of the relationship between body weight loss and baseline leptin level was plotted (linear regression coefficient: -0.067; correlation coefficient: -0.50; p = 0.098). Based on the linear regression model, weight loss by ≥7% is predicted when the blood level of leptin does not exceed 117.5 ng/ml. Nevertheless, more observations are necessary for the plotting of the linear model.

#### Types of eating behaviors

The type of eating behaviors was not different between the responders and non responders (p = 0.866, restrictive type; p = 0.856, external type; p = 0.361, emotional type).

#### Assessment of hunger/satiety on VAS

The factors of responses to appetite questions were evaluated separately for patients with obesity receiving liraglutide at 3.0 mg/day.

The response to the question, ‘How sated do you feel?’ was not a significant predictor of weight-reducing response (p = 0.064); however, the correlation coefficient was 0.51 (linear regression coefficient, 0.88). The response to the question, ‘How much food could you eat now?’, was not a significant predictor of weight-reducing response (p = 0.097), but the correlation coefficient was -0.46 (linear regression coefficient: -0.96).

The response to the question, ‘How much do you want to eat now?’, was not a significant factor associated with weight-reducing response (p = 0.300; correlation coefficient: -030).

#### Summary

Based on the obtained data, the significant factor in the prediction of response to therapy was the liraglutide dose (3.0 mg/day, dose-dependent efficacy), and for patients with obesity, the significant factor was baseline GLP-1 level in the blood (>2.15 ng/ml). With regard to our data and previously published data, BMI of 30–46 kg/m^2^ can be considered a predictor of high treatment efficacy with higher BMI values. Patients with a higher leptin level in the blood responded less to the therapy; the model should be plotted on the higher number of observations, and the predetermined cut-off value was 117.5 ng/ml. The responses to appetite questions were evaluated in a small number of patients, and the relationship between the factors and weight-reducing efficacy of liraglutide should be checked on a larger sample. Given the limited sample size, nonparametric statistical tests were used. These tests allow obtaining valid results even in a limited sample.

### Factors associated with a higher hypoglycemic efficacy

Hypoglycemic efficacy was determined as the decrease of HbA1c by ≥1.00% and absolute HbA1c decrease.

**Figure 2.**
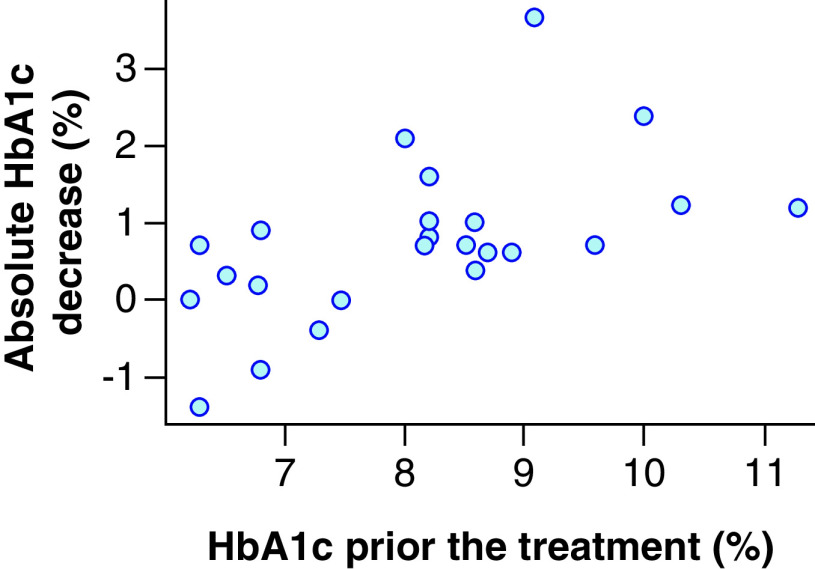
Relationship between an absolute hemoglobin A1c decrease and hemoglobin A1c prior the treatment.

**Figure 3.**
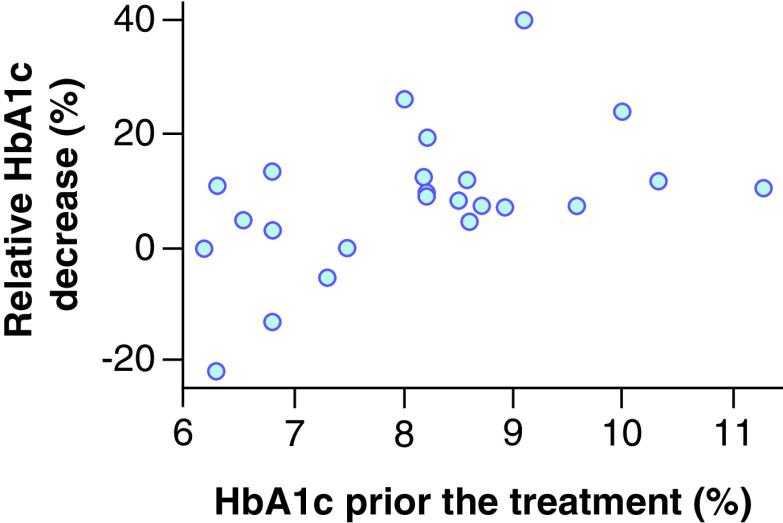
Relationship between a relative hemoglobin A1c decrease and hemoglobin A1c prior the treatment.

Patients whose HbA1c level was reduced by ≥1.00% as a result of the treatment had higher baseline values (9.21 ± 1.20%) than patients with lower HbA1c decrease (7.64 ± 1.05%; p = 0.012). The linear correlation of absolute HbA1c decrease depending on its baseline level, based on our data, are described by the following parameters: intercept, -2.75; linear regression coefficient: 0.43; correlation coefficient: 0.56 (p = 0.004). Therefore, the increase in the baseline level of glycated hemoglobin by 1% is associated with the additional predicted value decrease by 0.43% in 6 months. Figure 2 illustrates the relationship between the absolute decreases of HbA1c value depending on its baseline level. Figure 3 presents the relationship between the relative decreases (percentage of baseline level) of HbA1c values depending on the baseline levels. Our data show that the relative decrease is linear and reflects considerably the absolute decrease despite the varying baseline HbA1c values. Patients with baseline HbA1c value of at least 8.0% will receive the greatest benefit from liraglutide in relation to hypoglycemic drug efficacy.

When effective treatment response was evaluated as ‘HbA1c decrease by 1.00%’, other baseline parameters were not significant. Responders and non responders did not differ by age (p = 0.613), body weight (p = 0.690), BMI (p = 0.979), fasting glycaemia (p = 0.076), insulin (p = 0.143) and C-peptide (p = 1.000) levels in the blood, as well as GLP-1 (p = 0.493) and GIP (p = 0.442) levels in the blood. VAS values (satiety, desire to eat and amount of food which could be eaten by a patient) were not significant factors for the prediction of hypoglycemic response (p = 0.554; p = 0.267 and p = 0.214).

## Discussion

Tikhonenko *et al.* (2019) obtained quite different data, that is, patients having achieved weight loss ≥5% has higher BMI at baseline (p = 0.028) and the GLP-1 level was lower (p = 0.036) [7]. Their data contradict the results of our study. We supposed that their results were due to the inclusion of patients with T2DM only, with a mixed population having impaired glucose tolerance and T2DM. Furthermore, the T2DM and non-T2DM groups have significantly different GLP-1 levels, as shown in the present study: in patients with obesity but without the established diagnosis of T2DM, the GLP-1 level was higher than that in healthy individuals and the values in patients with obesity and T2DM were lower than those in healthy individuals. This result may be explained by the depletion of glucose reserve [20] and/or hyperglycemia in patients withT2DM; thus, GLP-1 synthesis was inhibited [13]. In the non-T2DM group with obesity, the level of leptin in the blood significantly correlated with the treatment response; the mean leptin level in the blood of responders (73.05 ng/ml) was lower than that in non responders (125.99 ng/ml; p = 0.018). These results support the idea, that desensitization of leptin receptors and the development of leptin resistance with impaired regulation of hunger and satiety is present in obese patients, owing to chronic hyperleptinemia [21]. This explains the observed less weight reduction in patients with a higher level of a leptin.

Nathan *et al.* (2016) also showed that response predictors to exenatide treatment were the high appetite level at the start of the therapy and female sex [22]. BMI, patient age and adverse events were not associated with the treatment response. The limitation of this study was the administration of exenatide only and the participants were adolescents. The only hypoglycemic efficacy predictor was the baseline HbA1c level, which is in line with previously obtained data [23].

The present study is exploratory, and it is limited by the small sample size and missing data. Further studies are needed to confirm the presented findings and to check the significance of the revealed correlations.

## Conclusion

Lower leptin and higher GLP-1 concentrations might predict better weight loss response to liraglutide. Liraglutide is probably more effective in patients with BMI 30–45 kg/m^2^.

## Future perspective

Identifying predictors of response to therapy as part of personalized medicine is a perspective future approach, which will reduce the time and cost to achieve a meaningful outcome in patient care.

**Summary points**

- Treatment of obesity leads to reduction of type 2 diabetes mellitus and cardiovascular risks and improvement of metabolic control and quality of life. Complementary body weight reduction, improvement of glycemic control and the correction of poor eating behaviors is usually needed.
- The response was more often observed in patients taking liraglutide at 3.0 mg/day (p = 0.007), that reflects the dose-dependent therapeutic effect.
- BMI is a significant predictor of response to GLP-1RA therapy. GLP-1RAs can be effective in patients with BMI 30–45 kg/m^2^.
- Patients with baseline hemoglobin A1c value of at least 8.0% will receive the greatest antiyhyperglycemic benefit from liraglutide administration.
- In non-type 2 diabetes mellitus group with obesity, the significant factor associated with the treatment response was the level of leptin in the blood; the mean leptin level in the blood of responders (73.05 ng/ml) was lower than that in non responders (125.99 ng/ml) (p = 0.018).
- Baseline GLP-1 level positively correlates with weight reduction after liraglutide therapy (3.0 mg/day) in patients with obesity.
